# Valuing health and wellbeing using discrete choice experiment: exploring feasibility, design effect and international preference similarity

**DOI:** 10.1007/s10198-025-01821-3

**Published:** 2025-07-30

**Authors:** Haode Wang, Donna L. Rowen, Yuen Chen, Clara Mukuria, Deborah Street, Richard Norman

**Affiliations:** 1 Sheffield Centre for Health and Related Research (ScHARR), The University of Sheffield, 30 Regent St, Sheffield City Centre, Sheffield, S1 4DA United Kingdom; 2 Centre for Health Economics Research and Evaluation (CHERE), University of Technology Sydney, Sidney, Australia; 3 School of Population Health, Curtin University, Perth, Australia

**Keywords:** DCE, Health-related quality-of-life, EQ-HWB, Valuation, QALYs

## Abstract

**Background:**

Discrete choice experiments (DCEs) are increasingly used in health preference elicitation studies. However, few studies have explored applying a DCE to value long health and wellbeing measures. This study evaluates feasibility, examines the impact of attribute ordering and explores if similar preference exists between countries.

**Methods:**

A health and wellbeing classification system was derived from the EQ Health and Wellbeing (EQ-HWB) measure based on dimensionality, item performance, stakeholder preference and cultural feasibility. Representative samples of UK and Australian general population completed 13 DCE_TTO_ tasks. Feasibility was assessed using data quality, time spent on the survey and each task, logical consistency and respondent understanding. Data were modelled using conditional logit model, to evaluate feasibility and impact of attribute ordering (health or other attributes ordered first). The UK and Australian value sets were compared on key characteristics, such as the relative importance of attributes, value set length and distribution.

**Results:**

2489 UK and Australian general public respondents completed the online DCE_TTO_ survey. Participants reported good understanding of the DCE_TTO_ questions and the attributes. Most of the more severe dimension levels had increasing disutility, with a higher proportion of insignificance observed with the wellbeing attributes. Physical health attributes had larger disutility than other attributes, with anchored utility values ranging from − 0.791 to − 0.588 to 1 for UK and Australian population. The preference between the two countries differed, with mixed evidence for ordering effects.

**Conclusions:**

DCE_TTO_ is a viable method for health and wellbeing preference valuation. However, health and wellbeing preference can be influenced by attribute ordering and national setting. The results have implications for the development of future health and wellbeing valuation studies.

**Supplementary Information:**

The online version contains supplementary material available at 10.1007/s10198-025-01821-3.

## Introduction

Recent valuation works have shown a growing interest in using discrete choice experiments (DCEs) to value health states [1–3]. DCEs are based on random utility theory which assumes that respondents make preference choices based on the presented attribute information but also contingent on unobservable random error [4]. A commonly used method is to anchor estimated latent values onto the 0 to 1 scale by trading time with health (also named DCE_TTO_) [5].

A number of existing generic health-related quality of life (HRQoL) measures, such as EQ-5D-5L^™^ has been valued using DCE. Existing valuation studies often focus on instruments with health-related attributes [3]. However, instruments encompassing a broader range of dimensions to capture health and wellbeing changes, like EQ Health and Wellbeing^™^ (EQ-HWB^™^), are less valued. EQ-HWB has two versions: the EQ-HWB-9 (previously EQ-HWB short) is a nine-item version consisting of the core domains, while EQ-HWB describes the longer full profile with 25 items [6]. There is potential demand for generating QALYs with a coverage extended beyond health attributes, yet there is no recommended valuation method [7].

Despite widespread use of DCE_TTO_ in eliciting health preferences, there remain critical gaps in our understanding when applying it to long health and wellbeing measure valuation. First, valuing a large number of attributes can impose a significant cognitive burden on respondents and may lead to issues of reliability and validity in the derived utility weights. Although an exploratory study investigated the feasibility of health and social care preference valuation (using EQ-5D and ASCOT measures) [5], the outcome was neither anchored onto a 0 to 1 scale, nor was the descriptive system generated by a single HWB measure [8]. An additional concern in the valuation of multi-attribute instruments is the potential influence of information presentation, especially attribute ordering [9]. Some studies have reported that the sequence in which attributes are presented can affect the choices made by respondents, but evidence remains inconclusive. For example, Mulhern et al. (2017) and Norman et al. (2016) did not observe consistent effects [10, 11], while other studies found relative prominence of attributes may shift depending on their position within the choice task [12, 13]. Given that many health state valuation studies rely on the assumption that respondents process each attribute independently of its position, a deeper examination of ordering effects with the health and wellbeing measure is crucial for future study design. Furthermore, cross-country comparisons of health state values are essential for understanding the generalization of valuation methods. Cultural differences, variations in healthcare systems, and diverse socio-demographic profiles can all influence how health states are valued. For example, distinct preference patterns were identified for several areas but the health attribute preference in UK and Australia shared similar characteristics [14–16]. This study seeks to determine, with the same valuation approach, whether the preference similarity is observed for health and wellbeing.

Although the three aims of this study may initially appear disparate, they are in fact integral components of the health and wellbeing measure valuation design. Assessing feasibility lays the foundation for further methodological development, where investigating attribute ordering effects quantify influence factors on respondents’ choices and the derived utility values. Comparing the results across the UK and Australia not only tests the preference generalization across culturally similar yet distinct populations, but also informs potential applications of prior information in multi-country settings. Therefore, the evidence ensures methodological robustness and feasibility relevant for any research targeting on valuing health and wellbeing measures. This study aims to systematically explore the three gaps mentioned, with a large sample. The research not only addresses the practical challenges but also contributes to the broader literature of valuing an extended health and wellbeing instrument using DCE_TTO_.

## Method

### Descriptive system

This study used EQ-HWB (2022 UK Experimental Version 1.0), which had seven domains (physical sensation, feelings and emotions, activity, self-worth, control and coping, relationship, cognition) [6] and 25 attributes, each with 5 levels of severity (No difficulty, Slight difficulty, Some difficulty, A lot of difficulty, Unable) or frequency (None of the time, Only occasionally, Sometimes, Often, Most or all of the time) (see Appendix I). However, as valuing the full 25 EQ-HWB attributes in a DCE task can be cognitively demanding and some attributes appear to be correlated to some extent [8], it is important to systematically select a subset of attributes that are collectively amenable to DCE valuation.

The attribute selection built upon item psychometric evidence generated by prior research on the EQ-HWB and conducted an independent evaluation of the items [17, 18]. The initial consideration of the nine EQ-HWB-9 items was based on existing research, while the systematic evaluation of the remaining 16 EQ-HWB items was undertaken as part of this study [6]. Specifically, we reassessed dimensionality, psychometric performance, stakeholder preferences, and cross-cultural performance using previously published evidence from EQ-HWB development and validation [6, 19–21] (Supplementary Material 1). This approach ensured that all of the available evidence was considered in the item re-selection process, and the chosen attributes were both methodologically robust and practical for valuation purposes.

A qualitative component was incorporated into this study to ensure the clarity and appropriateness of the selected attributes for valuation. We conducted cognitive interviews and focus groups with a sample of potential respondents (*n* = 11) from the general population in the UK to assess how well they understood the survey wording and whether any attributes required refinement. These participants were purposively recruited to represent a diverse mix in terms of age, gender, and educational background. In each focus group, participants were provided with the sample DCE_TTO_ task and various instruction wordings. They were then asked a series of structured questions aimed at evaluating clarity and interpretation of attribute information, instructions for the DCE_TTO_ tasks, and discuss the relevance of different dimensions in decision-making. The insights gathered from this qualitative work informed refinements in attribute introduction but did not necessitate changes to the items and levels wording. This qualitative validation was necessary for strengthening the feasibility of the final attribute set for use in the DCE_TTO_ tasks. More details for the qualitative consultation are reported in Supplementary Material 2.

### DCE valuation tasks

A DCE_TTO_ task involved two health profile options described by the selected level of each attribute and the duration of health state. All attributes that varied between options were highlighted to promote engagement. To evaluate the impact of information order, we designed two valuation task formats: a health-first DCE_TTO_ and wellbeing-first DCE_TTO_. In the health-first design, attributes primarily associated with physical and mental health were presented first, followed by attributes linked to broader wellbeing aspects (Supplementary Material 3A). The wellbeing-first design reversed this order (Supplementary Material 3B). Attributes were classified based on the dimensions and qualitative consultation. Health attributes were defined as those directly related to physical function (e.g., mobility, pain severity) and mental health symptoms (e.g., anxiety, depression), whereas wellbeing attributes encompassed broader social, emotional, mental health and life satisfaction aspects (e.g., loneliness, feeling unsupported, control), overlapping with aspects of health. We considered the uncertainty for attribute interpretation and classification decisions were refined through qualitative consultations with respondents, by asking the focus group participants whether the given attribute is more related with “health” or “wellbeing”. ‘Physical pain’ was categorized as a health attribute but appeared at the bottom in all tasks due to balancing considerations.

HWB information in each choice pair remained the same for both designs. An initial literature review [3] was conducted to support the selection of duration attribute levels.

### Selecting choice tasks

The number of possible scenarios was too large for all combinations to be valued, meaning a subset of combinations needed to be selected. To reduce cognitive burden and enhance comparability, controlled overlap was imposed on half of the attributes [22]. The total number of choice sets had to exceed the number of parameters (greater than 13 × 4 + 1 = 53 for DCE_TTO_ design) [23, 24]. An orthogonal design was employed and 600 choice sets were generated using generator approach (6 generators × an orthogonal array of size 100) [9, 24]. The selection of overlapping attributes accounted for information saturation, with half of the attribute levels in each generator changed. Two choice sets from each generator (2 × 6 = 12 choice sets for each block, 50 blocks in total) were selected for each block [25]. One extra choice set from the 600 pairs was adjoined to each of the 50 blocks, ensuring each block had one dominated choice set and the attribute levels were more balanced. Each DCE_TTO_ block had 13 choice sets. The aggregated number of observations provides robust statistical power for estimating conditional logit models [7].

The design was generated using Wolfram Mathematica software (version 13.3) and R software (version 4.3.1), which is widely used for constructing efficient choice experiments [9].

### Survey administration

The online survey began with information about the project and survey, followed by a digital informed consent page. The survey had 4 parts. First, respondents completed demographic questions, health questions (including selected EQ-HWB attributes) about disability/activity limitations and general health, and one repeated employment status question as an attention check question.

Second, respondents were randomly allocated to one of the two study arms until the target sample size was saturated. Respondents were shown instructions about the task and one practice DCE_TTO_ that explained the task. Respondents then completed 13 DCE_TTO_ tasks in a random order. Subsequently, respondents were asked a series of follow-up questions designed to explore their decision strategy and stated attribute importance. For the decision strategy question, respondents were asked about their cognitive strategies that participants used when making the DCE_TTO_ choices. An attribute importance question sought to identify the five most important attributes in their evaluations.

Finally, 5 questions about the engagement, understanding and difficulty of the whole survey were presented, along with a free-text question for any general feedback (Supplementary Material 4).

### Recruitment and participants

Participants (n=100) were recruited in the two countries online to pilot the survey in early 2024. The main survey aimed to recruit quota samples [14], using education level, age and gender [3, 26, 27], from the UK and Australian general population. Respondents were recruited through an online panel by a market research agency (SurveyEngine) with a target sample size of 1200 in each country. The inclusion criteria were adult, English speaking local resident and had qualified equipment (large screen equipment) to complete the survey. Those who completed the whole survey received small incentives. Responses with repeated IP or that failed the automated program check were excluded. Ethical approval to conduct this research was granted in the UK by the University of Sheffield (approval number: 051907) and in Australia by Curtin University (approval number: HRE2024-0055).

### Data analysis

All data were analyzed separately by country and attribute ordering (health-first vs. wellbeing-first). The DCE_TTO_ data were analyzed using a conditional logit fixed-effects model [28]. The model specification is [29, 30]:

1 $$\:{U}_{ij}={\beta\:}_{1}{t}_{ij}+{\stackrel{-}{\beta\:^{\prime}}}_{2}{X}_{ij}\times\:{t}_{ij}+{\varepsilon}_{ij}$$

where the $\:{U}_{ij}$ represents the utility of individual *i* for the health state *j*, assuming that neither the order of presentation nor the specific health state influences the utility of *j*. $\:{\epsilon\:}_{ij}$ represents the error term, $\:{\beta\:}_{1}$ and $\:\stackrel{-}{{\beta\:}_{2}}\:$represent the coefficients for the duration in life years *t* and the coefficients for the dummy-coded severity levels multiplied by duration, respectively, with level 1 (the best health attribute level) serving as the baseline for each attribute. $\:{\beta\:}_{1}$ represents the value respondent *i* assigns to living in perfect health for 1 year. The latent coefficients are anchored using the coefficient of duration $\:{\beta\:}_{1}$ (i.e. the marginal rate of substitution):

2 $$\:{\beta\:}_{2}=\frac{\stackrel{-}{{\beta\:}_{2}}}{{\beta\:}_{1}}$$

Attribute-level interactions were not considered as we had no prior hypothesis regarding interactive terms, and the number of possible combinations was too large. Model estimation was conducted using Stata version 17. We conducted different data analyses for the three objectives:

#### Objective 1: feasibility of DCE

The feasibility assessment considered both respondent preferences and data quality. First, we examined completion time, attention check responses, self-reported decision strategies, and respondent feedback. Completion time thresholds were applied, where responses with a total survey duration of less than 10 min or any DCE_TTO_ task completed in under 10 seconds were flagged as potential speeders.

Second, we assessed modelling feasibility by evaluating the proportion of logically ordered and statistically significant anchored coefficients. Ideally, attribute-level coefficients should reflect a disutility for each health impairment relative to the baseline level and exhibit higher (or equal) disutility for more severe adjacent levels, maintaining monotonicity.

Third, we compared the relative importance of modelled coefficients from choice data with stated attribute importance. The ranking of modelled attributes was determined based on the relative importance of the worst-level disutility [31], where the highest-ranked attribute corresponded to the greatest utility decrement at its most severe level [14]. Stated attribute importance is derived from the questionnaire follow-up question.

Finally, we examined the feedbacks of respondents.

#### Objective 2: information order effect

The impact of attribute ordering was evaluated by comparing attribute coefficients across designs, examining the preference order of attributes, and analyzing the distribution of utilities [14]. A Wald test, based on the Hessian matrix, was used to assess differences in preferences between designs [16, 32]. Additionally, a Swait-Louviere test was conducted to assess differences related to scale heterogeneity [33, 34], with significance determined at P value thresholds of 0.05 and 0.1. We also examined the distribution of utility values by its range and state values.

A pooled conditional regression was also estimated, incorporating data from both designs. The pooled data modeling included all level coefficients and interaction terms for order effects. The attribute order dummy variable $\:Order$ was coded as 0 if the choice set followed the health-first order and 1 if it followed the reversed order. The utility function is as follows:

3 $$\eqalign{ {U_{ij}} & = {\beta _1}{t_{ij}} + {\beta _1}{t_{ij}} \times Order + \beta _2^\prime {X_{ij}} \times {t_{ij}} \cr & + \beta _2^\prime {X_{ij}} \times {t_{ij}} \times Order + {\varepsilon _{ij}} \cr}$$

A significant order effect term $\:{\beta\:}_{1}{t}_{ij}\times\:Order$ would indicate that respondents’ preferences were influenced by the presentation order of the attributes.

#### Objective 3: UK and Australia preference differences

We compared the UK and Australian anchored coefficients from the health-first DCE_TTO_ data to assess whether preferences were similar between the two country samples, following an approach similar to the EQ-5D value set comparison study [30]. However, level coefficients were not directly compared, as variations in sample quotas introduced additional confounding factors. The variance in health state utility was examined using a two-way random-effects intraclass correlation coefficient (ICC) and Pearson’s correlation. Variance levels were interpreted as high (< 0.5), moderate (0.5–0.75), and low (> 0.75).

## Results

### Classification system

Exclusion decisions were made with several EQ-HWB items: Frustrated, Support, Memory, Stigma/belonging, Unsafe, Discomfort severity, and Enjoyable activities were excluded due to strong correlation with EQ-HWB-9 attributes and/or low overall validity. The Pain frequency and Discomfort severity attribute was dropped due to strong correlation with other pain and discomfort items. The decision to exclude Hopeless and Coping was driven by EQ-HWB stakeholder preference [6] and correlation results. Self-worth was excluded due to worse factor analysis performance.

Thirteen items met the selection criteria—including all nine EQ-HWB-9 items along with four additional items—and were included as DCE_TTO_ attributes: Vision, Hearing, Mobility, Daily activity, Control, Concentrating/ thinking clearly, Anxiety, Sad/depressed, Loneliness, Support, Sleep, Fatigue, Pain severity (Table 1 and Supplementary Material 1). The duration levels considered were 1, 4, 5, 7, and 10 years [28].

### Design finalization and pilot

Eleven participants took part in four focus groups. The EQ-HWB information was found to be interpretable, and participants were able to understand and complete the DCE_TTO_ tasks independently.

The initial pilot study collected 100 responses (50 per country), of which 97 were deemed valid. The average time to complete one DCE_TTO_ question was 20 s. Participant feedback indicated that the survey functioned effectively, and no modifications were made following the pilot phase.

### Sample and data

A total of 2,489 respondents completed the survey, with 26 excluded due to repeated IP addresses or bot detection. The final analysis included 2,463 respondents (1,227 from the UK and 1,236 from Australia).

**Fig. 1:**
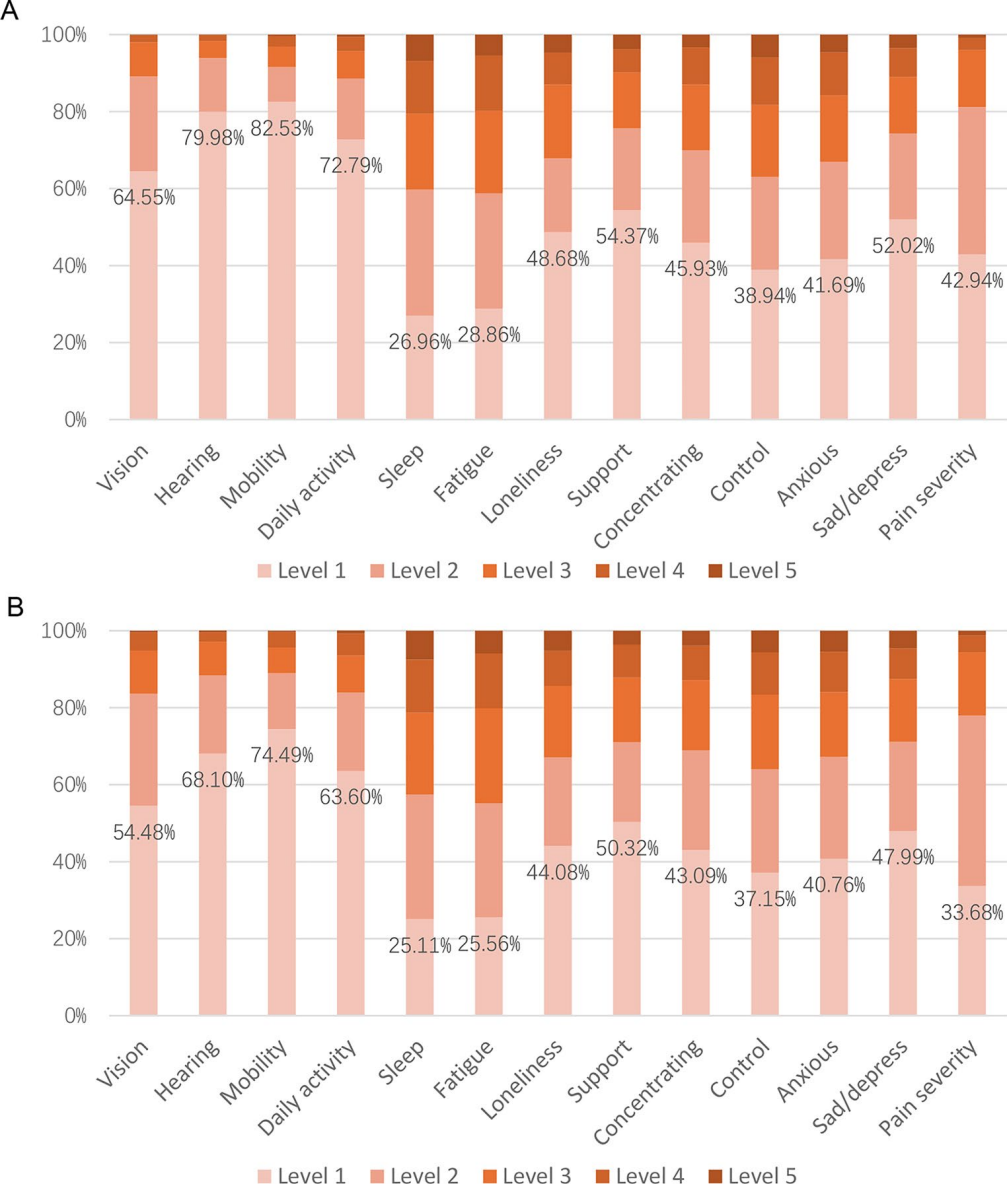
Self-reported EQ-HWB 13 attribute conditions. **A**. UK sample responses to the items. **B**. Australian sample responses to the items

Socio-demographic information is presented in Table 2. The average age of respondents was 47.3 years, and approximately half were male. In the UK sample, over 14% were informal carers, while 7% were receiving care from an informal carer. Sample demographics were generally well balanced across study designs and comparable to the general population, except that respondents in the health-first DCE_TTO_ group were slightly older and included a higher proportion of retired individuals. Overall, 27% of respondents reported limitations in day-to-day activities, and 24% classified their health as fair or poor (Fig. 1).

The Australian sample was older and more highly educated than the general population. The average age was 48.2 years, compared to 40.7 years in the general population, and 43% had a bachelor’s degree or higher, compared to 29% in the general population. Approximately one-quarter of respondents were informal carers, and 14% received care from others, a higher proportion than in the UK sample. Sample demographics were well balanced across groups. Similar to the UK sample, 76% of respondents reported good or better health. However, the proportion reporting some level of limitation in daily activities (36%) was higher than in the UK sample (Fig. 1).

### Feasibility

**Fig. 2:**
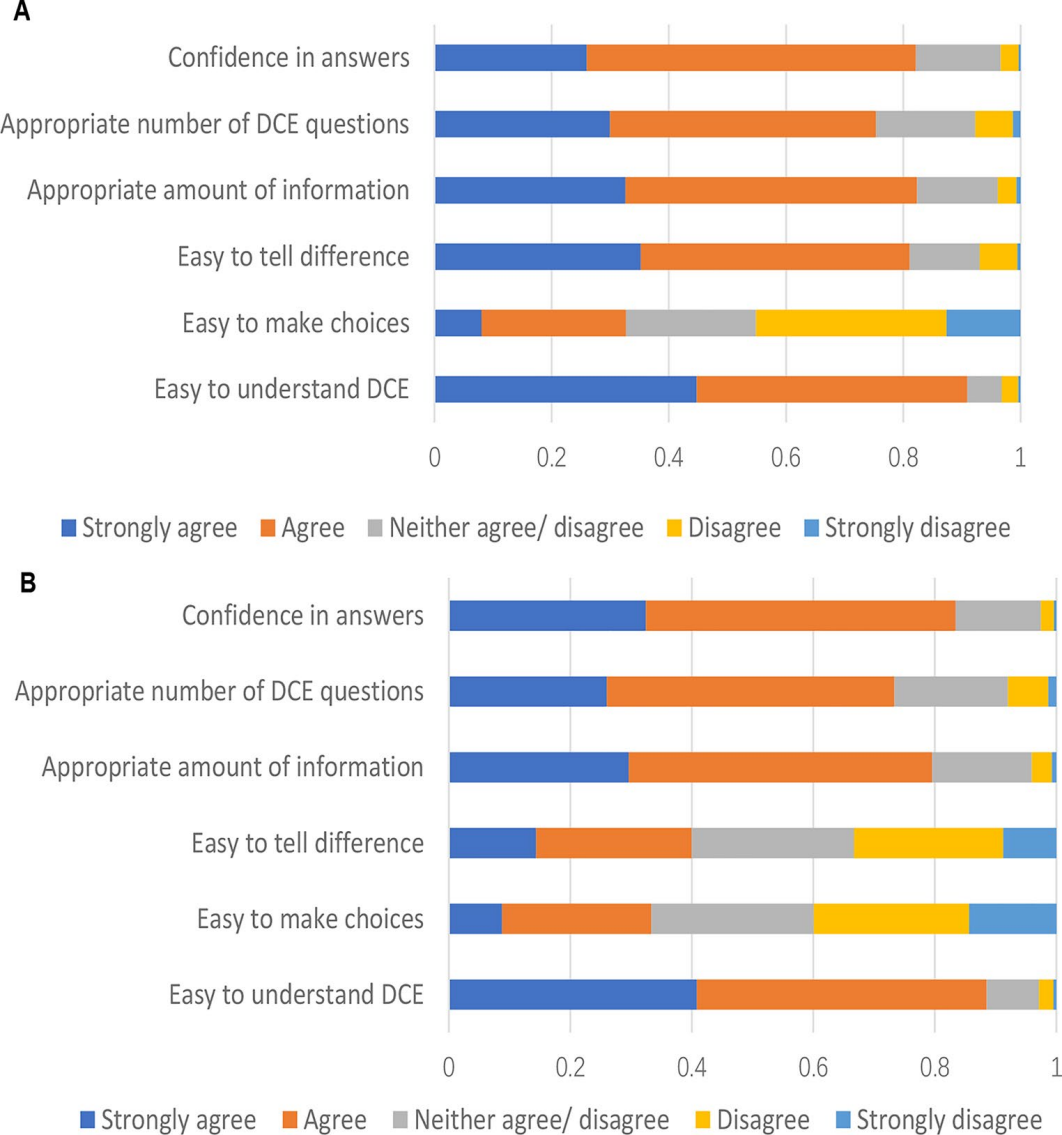
Survey feedback. **A**. Survey feedback from UK sample. **B**. Survey feedback from Australian sample

The median survey completion times were 16.46 (S.D.12.67) and 18.69 (S.D. 12.99) minutes for the UK and Australian samples respectively. The proportion of speeders in both countries was higher than 25%. In the perspective of decision strategies applied, over 27% of all respondents considered all of the health and wellbeing information all the time. However, 18% of all respondents focused on the duration of the state. 1.5–2.3% of respondents in each cohort did not provide consistent answer for the repeated career question. Most of the UK and Australian respondents (over 80%) felt confident with their choices, agreed that the number of choices sets per person was appropriate and that the amount of information provided was appropriate. However, around 15% of all respondents found it hard to tell the difference and/or make the choice in the two countries (Fig. 2).

All models were statistically significant at the 5% significance level. The marginal effects demonstrated steeper utility declines between levels 3 and 4, as well as between levels 4 and 5. Most coefficients were statistically significant and followed a monotonic pattern across both attribute order designs at the 5% significance level. However, some evidence of non-significant coefficients, positive disutility, and non-monotonicity was observed. Instances of non-monotonicity and insignificance were primarily associated with milder severity levels (levels 2 and 3). Health attributes such as Sleep and Anxiety, along with wellbeing attributes—including Loneliness, Support (in the Australian data), and Control (in the UK data)—had the highest number of non-significant and/or non-monotonic or positive level coefficients (Table 3).

Utility values for HWB states were calculated by summing the utility decrements and adding the result to 1. The range of disutility values spanned from 1 (state 1111111111111) to -0.634 (state 5555555555555) for the UK sample and from 1 to -0.539 for the Australian sample (Table 3).

The top-five ranked attributes, by its worst level disutility, were predominantly physical health attributes: Pain Severity, Vision, Daily Activity, and Mobility (identified across all models), as well as Hearing (excluded in all models with wellbeing-first DCE_TTO_ data). Anxiety, a mental health attribute, was ranked in the top-five only in models with health-first DCE_TTO_ data). The stated attribute ranking was based on a clustering of each attribute being selected as the most important attribute. Rankings from regression analysis and stated preferences were compared across study designs, demonstrating a high degree of consistency (Supplementary Material 5A).

Most respondents from both the UK and Australia expressed confidence in their choices and agreed that the number of choices sets per person and the amount of information provided were appropriate. However, approximately 15% of respondents in both countries reported difficulty in distinguishing between options and/or making a decision (Fig. 2).

### Attribute ordering effect

In the health-first DCE_TTO_, only one significant non-monotonic level coefficient was identified in both the UK and Australian samples. In contrast, the wellbeing-first DCE_TTO_ exhibited three significant non-monotonic coefficients in the UK sample and five in the Australian sample. The Wald test results indicated that 4 out of 78 significant level coefficients (attributes are Hearing, Mobility and Sleeping) differed significantly at the 5% level (Table 4). The Swait-Louviere test found no significant differences related to scale heterogeneity.

Regression analysis using the pooled dataset suggested the presence of an ordering effect with some of the attribute levels. In the UK sample, ordering effects were significantly positive (indicating smaller disutility) for Sleep and Depression levels, whereas they were significantly negative (indicating larger disutility) for Hearing, Mobility, and Control levels. In the Australian sample, ordering effects were significantly positive for time, suggesting that duration was weighted more in the wellbeing-first design. Hearing, Mobility, Concentration, and Control levels exhibited a significant negative ordering effect (Supplementary Material 5B).

An opposite trend in health state utility distributions was observed between the UK and Australian samples. In the UK data, utility values for mild and moderate health states (from perfect health to state 4444444444444) were higher in regression with wellbeing-first data compared to health-first data, whereas extreme state values (from state 4444444444444 to state 5555555555555) were lower. The wellbeing-first DCE_TTO_ generated wider overall range of utility value (-0.791 versus − 0.634). Conversely, in the Australian sample, utility values for mild health states (from state 2222222222222 to state 3333333333333) were lower in wellbeing-first data compared to health-first data, while moderate to worst state utility values (from state 4444444444444) were higher. The health-first DCE_TTO_ had larger size of value decrements with the worst health state than the wellbeing-first DCE_TTO_ (-0.588 versus − 0.539) (Table 3).

### UK and Australian sample preference difference

While some minor differences in preferences were identified, there were striking similarities across the two countries. Both the UK and Australian samples ranked Vision, Hearing, Mobility, and Pain Severity as the most important attributes. The utility values of 244 selected health states, 240 selected using D-efficient criteria and 4 benchmark states (2222222222222 to 5555555555555), were compared across the two value sets (Supplementary Material 6), revealing a consistent trend in utility movements. Differences in utility values became more pronounced for more severe health states (i.e., those worse than 4444444444444). ICC analysis attributed less than 5% of the variance to differences in sample preferences for both designs. Additionally, the Pearson’s correlation coefficient was 0.96, indicating that health state values from the two samples followed an almost linear relationship and the variance was extremely low.

## Discussion

Our study provides empirical evidence on the application of the DCE_TTO_ method for valuing a long health and wellbeing measure. The feasibility evaluation confirms that, despite the measure complexity, health and wellbeing measure can be effectively valued using a DCE_TTO_ method. That the majority of participants in four groups found the DCE_TTO_ questions easy to understand, easy to tell the difference, confident with their answers, and spending a reasonable amount of time is a positive indicator that the DCE_TTO_ elicited preference is reliable.

Although DCE_TTO_ method is generally feasible, careful attention to study design is essential. Our study found that 13–20% of respondents reported some difficulty in making choices and/or distinguishing between options. These figures must be interpreted in the context that changes in wellbeing are influenced not only by actual differences but also by factors such as adaptation, individual circumstances, and response scale effects [35]. As the number of attributes and dimensions increases, the level of perceived difficulty in decision-making is also expected to rise. Evidence of heuristic decision-making—where over 20% of respondents primarily considered the duration of the health state when making trade-offs—suggests that the cognitive burden associated with processing large amounts of information may have contributed to decision-making challenges. However, heuristics in choice tasks are relatively common, and the implications of heuristic-driven responses remain a subject of debate [36, 37].

Regression coefficients across levels were generally logically consistent, though a larger proportion of wellbeing-related or mild-level coefficients were found to be insignificant and/or non-monotonic. Attribute levels with significant but non-monotonic values may indicate potential comprehension issues [38]. Six attributes—Mobility, Sleep, Loneliness, Support, Fatigue, and Anxiety—exhibited significant but non-monotonic or positive coefficients at frequency levels 2 (“Only occasionally”) and 5 (“Most or all of the time”) across the four models. One potential option to consider is to present the numerical level information (e.g., 1 to 5 for the levels) to help respondents “decipher” the descriptive levels [39]. While Mattmann et al. have explored the role of sociodemographic factors in influencing decision certainty [40], our study provides insights into how the attributes of health and wellbeing measures themselves may contribute to decision stability. The high consistency between the top-five ranked attributes in the stated and regression-based rankings suggests that respondents demonstrated a high level of certainty and consistency in their decision-making. Despite some reported difficulty and level coefficient insignificance/non-monotonicity, the overall evidence—including reasonable survey completion times, a high proportion of logically consistent responses, respondent feedback and the attribute order consistency—supports the conclusion that the DCE_TTO_ method is feasible for valuing an extended health and wellbeing measure.

From the perspective of information ordering, this study presents mixed evidence. The Wald test and pooled regression with interaction terms suggest that Hearing, Mobility, Sleep, Depression, Control and time exhibited significant order effects, though the disutility magnitude was small (anchored disutility < 0.1). The ordering effect on clusters of dimensions had a limited impact on attribute preferences, consistent with findings from other studies assessing ordering effects in health-related quality of life measures [10, 11, 41]. However, differences were observed in the anchored utility distribution of health states, which may not be apparent when comparing single coefficients alone. This finding highlights the importance of considering broader distributional effects rather than focusing solely on individual parameter estimates. Future health and wellbeing valuation studies are recommended to adhere to the natural ordering of the measure —specifically, the order in which items are presented when the patient-reported outcome measure is self-completed, to increase the comparability and consistency of utility values.

There is a high degree of similarity in health state preferences between the UK and Australian samples, with both populations consistently ranking attributes such as vision, hearing, mobility, and pain severity as the most important, and a high ICC statistic found. This finding aligns with previous research on cross-country similarities in HRQoL measures [14] and the psychometric evaluation result [6]. Although local sample quotas were applied in both countries, the results have several important implications for future valuation study designs. First, they support the potential for transferring prior information between countries with similar cultural and healthcare contexts, which could enhance the efficiency of valuation study design. Second, the comparable health and wellbeing preference suggest that pooling data from countries like the UK and Australia may be justified, enabling multi-country analyses and potentially facilitating preference predictions across countries. However, while minor differences do not substantively alter overall trends, they do influence utility scaling, indicating that cost-effectiveness analyses would still benefit from using country-specific value sets to ensure accuracy and relevance.

As introduced in the methodology section, the descriptive system applied in this study “bolts-on” attributes to the EQ-HWB-9 measure. By comparing preferences of the overlapped attributes for the “bolt-on” descriptive system with preferences elicited from the same population using EQ-HWB-9, it is noteworthy that relative attribute importance had significant differences. The results from the health-first DCE_TTO_ data analysis indicate that respondents prioritized Pain Severity, Vision, Anxiety (UK only), Mobility, and Daily Activity as the most important attributes. In contrast, with the same overlapped attributes, preference analyses using DCE for the EQ-HWB-9 confirmed Pain Severity, Mobility, and Daily Activity as the most important but ranked Anxiety as the least important attribute [42, 43]. Similarly, Control was ranked as one of the lowest in this study, while it was ranked top 3 to 5 in the aforementioned studies. Another key difference lies in how the instruments conceptualize and weigh different domains. In the valuation studies of EQ-HWB-9 and EQ-5D-5L + ASCOT (which captures social-care-related quality of life), health-related attributes (e.g., mobility, pain severity) received weights comparable to those of broader quality-of-life attributes [5], though the ASCOT specifically reflects aspects of social care and should not be conflated with the more comprehensive health and wellbeing measures captured by EQ-HWB. In contrast, by adding critical physical function attributes (e.g., Vision and Hearing) in the descriptive system, our study found more pronounced differences in relative weights, with health-related attributes receiving greater weights. These findings have important implications for the further development and valuation of “bolt-on” health and wellbeing descriptive systems. This highlights the need for further research into more complex models that account for dimensional interactions. Moreover, these results diverge from earlier comparative studies, which found that adding a health attribute (Tiredness) did not significantly impact the mean value of moderate health states when using the using Visual Analogue Scale or Time Trade-Off methods [44, 45]. The discrepancy may stem from differences in the valued measure and valuation methodology, requiring additional research to explore the valuation methodology and measure implications.

There are a number of limitations with this study that future studies can avoid. First, data sampling can be improved. All participants were recruited from an existing panel, which raises potential concerns about data quality in online samples [46]. Despite quality control measures, completely excluding low-quality respondents or bots remains challenging. Second, the study design treated duration as a continuous variable to increase comparability with the published evidence, following classic methods, which may overlook the possibility of discrete time preferences [47]. Exploring hybrid approaches that account for discrete and continuous time preferences could provide further insights into preference heterogeneity. Thirdly, the order effect regression used a pooled dataset combining both designs, which could introduce scale heterogeneity. However, the primary objective of this pooled regression was to identify the significance of interaction terms rather than to compare attribute preferences. Notably, prior DCE_TTO_ valuation studies, such as those for the EQ-5D-5L measure, have found scale heterogeneity to be insignificant [38]. Lastly, modifications to the EQ-HWB measure were made after the completion of this study, including refinements to item wording and domain representation, as part of ongoing validation efforts. While these changes may affect the exact preference weights derived from this study, the findings on the feasibility of using DCEs for valuing extended health and wellbeing measures should be robust.

## Conclusion

The DCE_TTO_ is a feasible method for valuing a long health and wellbeing measure, successfully generating logically consistent preference weights for most attribute levels. While attribute ordering had a limited impact on attribute-level preferences, it influenced the distribution of utility values. Additionally, health and wellbeing preferences were largely similar between the UK and Australia. These findings have important practical implications for the design of future health state valuation studies, supporting the potential for cross-country comparisons and methodological refinements in health preference research.

**Table 1:** Thirteen attribute classification system for valuation

	Domain	Attribute	Levels				
			No difficulty	Slight difficulty	Some difficulty	A lot of difficulty	Unable
1	Vision	How difficult was it for you to see (using, for example, glasses or contact lenses if they are needed)?	□	□	□	□	□
2	Hearing	How difficult was it for you to hear (using hearing aids if you usually wear them)?	□	□	□	□	□
3	Mobility	How difficult was it for you to get around inside and outside (using any aids you usually use e.g., walking stick, frame or wheelchair)?	□	□	□	□	□
4	Daily activity	How difficult was it for you to do day-to-day activities (e.g., working, shopping, housework)?	□	□	□	□	□
5	Control		None of the time	Only occasionally	Some of the time	Often	Most or all of the time
		I felt I had no control over my day-to-day life (e.g., having the choice to do things or have things done for you as you like and when you want).	□	□	□	□	□
6	Concentrating/ thinking clearly	I had trouble concentrating/thinking clearly	□	□	□	□	□
7	Anxious	I felt anxious	□	□	□	□	□
8	Sad/depressed	I felt sad/depressed	□	□	□	□	□
9	Loneliness	I felt lonely	□	□	□	□	□
10	Support	I felt unsupported by people	□	□	□	□	□
11	Sleep	I had problems with my sleep	□	□	□	□	□
12	Fatigue	I felt exhausted	□	□	□	□	□
13	Pain severity	I had no physical pain in the last 7 days	□				
		I had mild physical pain in the last 7 days	□				
		I had moderate physical pain in the last 7 days	□				
		I had severe physical pain in the last 7 days	□				
		I had very severe physical pain in the last 7 days	□				

*Notes*: The ordering of the attributes has not been changed in comparison to the order of EQ-HWB. Information in domain column show the labels used in the following sessions to refer to these attributes

**Table 2:** DCE survey respondents of each cohort

Characteristics	UK general population^1^	Australian general population^1^	Cohort 1^2^ sample (*n* = 627), %	Cohort 2^2^ sample (*n* = 600), %	Cohort 3^2^ sample (*n* = 632), %	Cohort 4^2^ sample (*n* = 604), %	Overall
**Gender**							
Male	49%	49%	48.80%	48.00%	51.42%	50.99%	49.80%
Female	51%	51%	51.20%	51.83%	48.42%	48.68%	50.03%
Prefer not to say			0	0.17%	0.16%	0.33%	0.17%
**Age**							
Ave. age	40.70	40.70	47.47	46.92	48.56	47.96	47.73
**Education**							
Low education	27.80%	27.33%	0.48%	0.33%	12.82%	10.60%	6.06%
Medium education	35.60%	43.90%	41.47%	43.50%	44.46%	45.20%	43.66%
Higher education	34.00%	28.60%	57.74%	55.67%	42.25%	44.20%	49.97%
Prefer not to say			0.31%	0.16%	0.47%	0.00%	0.24%
**Median time**							
Total (seconds)			990.84	983.88	1148.64	1094.46	1054.46
S.D.			789.84	729.48	828.59	730.49	769.6
DCE question (seconds)			28.46	27.24	27.51	28.44	27.91
S.D.			39.56	28.08	27.68	27.79	30.78
**Employment**							
Full-time or Part-time employed			55.19%	57.66%	60.13%	61.10%	58.52%
Retired			22.81%	20.67%	22.15%	23.01%	22.16%
Student			6.22%	8.33%	3.80%	3.15%	5.38%
Unemployed			8.29%	4.83%	6.65%	6.79%	6.64%
Long-term sickness			3.35%	2.50%	2.37%	1.99%	2.55%
Look after family/home			3.19%	5.00%	2.85%	3.15%	3.55%
Prefer not to say			0.16%	0.17%	0.47%	0.00%	0.20%
Other			0.80%	0.83%	1.58%	0.83%	1.01%
**Care status**							
Carer for an adult(s) family member or friend (not as a paid job)			14.99%	14.67%	23.89%	25.33%	19.72%
Cared for by other adults (including paid carers) because of health or age			7.18%	7.17%	14.40%	14.40%	10.79%
Neither of the above			76.40%	77.33%	1.90%	0.83%	39.12%
Prefer not to say or don’t know			1.44%	0.83%	59.81%	59.44%	30.38%
**Day-to-day activities limitation**							
Yes, limited a lot			7.97%	6.67%	9.34%	9.77%	8.44%
Yes, limited a little			18.66%	21.17%	29.11%	24.67%	23.40%
No			72.57%	71.33%	60.76%	65.23%	67.47%
Prefer not to say			0.80%	0.83%	0.79%	0.33%	0.69%
**General health**							
Excellent			9.41%	11.83%	12.82%	13.74%	11.95%
Very good			33.17%	31.00%	26.11%	28.81%	29.77%
Good			31.26%	32.33%	34.97%	33.77%	33.08%
Fair			22.49%	20.67%	22.15%	18.54%	20.96%
Poor			3.67%	4.17%	3.96%	5.13%	4.23%

*Notes*: 9 UK and 13 Australian respondents did not provide the sex, (and/or) education, (and/or) marital status information

^1^The general population quota is from the UK Office for National Statistics (ONS) and Australian Institute of Health and Welfare

^2^Model explanation: Cohort 1: health-first DCE_TTO_ design with UK sample; Cohort 2: wellbeing-first DCE_TTO_ design with UK sample; Cohort 3: health-first DCE_TTO_ design with Australian sample; Cohort 4: wellbeing-first DCE_TTO_ design with Australian sample

**Table 3:** Conditional logit regression of DCE survey responses, by sample

Attribute	Cohort 1 UK sample health-first design	Cohort 2 UK sample wellbeing-first design	Cohort 3 Australian sample health-first design	Cohort 4 Australian sample wellbeing -first design
Seeing (anchored)				
2	-0.067	-0.059	-0.037*	-0.081
3	-0.096	-0.082	-0.039**	-0.087
4	-0.225	-0.175	-0.161	-0.148
5	-0.369	-0.340	-0.308	-0.258
Hearing (anchored)				
2	-0.046	-0.012**	-0.066	-0.064
3	-0.061	-0.047	-0.083*	-0.082
4	-0.097	-0.110	-0.101	-0.103
5	-0.120	-0.197	-0.139	-0.179
Mobility (anchored)				
2	-0.029*	-0.029**	-0.003**	-0.064
3	-0.044	0.003**^1^	-0.015**	-0.050^1^
4	-0.105	-0.082	-0.133	-0.085
5	-0.140	-0.203	-0.134	-0.203
Day to day activities (anchored)				
2	-0.018**	-0.016**	-0.064	-0.046
3	-0.062	-0.045	-0.033**^1^	-0.059
4	-0.112	-0.080	-0.101	-0.103
5	-0.168	-0.177	-0.164	-0.143
Sleeping (anchored)				
2	-0.020**	0.066^1^	0.002**^1^	0.051**^1^
3	-0.026**	0.031^1^	0.010**^1^	0.043*^1^
4	-0.049	-0.010**^1^	-0.021**	0.023**^1^
5	-0.037*^1^	-0.011**^1^	-0.021**	-0.027**
Exhausted (anchored)				
2	0.008**^1^	-0.013**	-0.007**	-0.020**
3	0.003**^1^	-0.055	-0.007**^1^	-0.060
4	-0.041	-0.084	-0.024**	-0.048^1^
5	-0.059	-0.030**^1^	-0.028**	-0.055
Lonely (anchored)				
2	0.001**	-0.009**	-0.010**	0.021**^1^
3	0.003**^1^	-0.005**^1^	-0.010**^1^	-0.005**
4	0.001^1^	-0.060	-0.051*	-0.043*
5	-0.036*	-0.052^1^	-0.051*	-0.009**^1^
Unsupported (anchored)				
2	-0.038*	-0.003**	-0.032**	-0.055
3	-0.050	-0.003**^1^	-0.053**	-0.028**^1^
4	-0.087	-0.039	-0.088**	-0.075
5	-0.029**^1^	-0.032*^1^	-0.043**^1^	-0.057^1^
Concentration (anchored)				
2	-0.044	0.012**^1^	0.006**^1^	-0.017
3	-0.082	-0.040	-0.031**	-0.022
4	-0.019**^1^	-0.059	0.007**^1^	-0.061
5	-0.055	-0.080	-0.025**	-0.030**^1^
Anxious (anchored)				
2	0.017**^1^	-0.006**	-0.017**	0.009**^1^
3	0.018**^1^	-0.020**	-0.015**^1^	-0.012**
4	-0.032**	-0.034**	-0.031**	-0.052
5	-0.038	-0.073	-0.060	-0.042^1^
Depression (anchored)				
2	-0.053	0.017**^1^	-0.014**	-0.006**
3	-0.032**^1^	-0.021**	0.006**^1^	-0.008**
4	-0.108	-0.059	-0.054*	-0.041*
5	-0.149	-0.097	-0.116	-0.052
Control (anchored)				
2	0.020**^1^	-0.044	0.008**^1^	-0.048
3	-0.013**	-0.024**^1^	-0.018**	-0.006**^1^
4	-0.011**^1^	-0.032	-0.028**	-0.039
5	-0.060	-0.069	-0.065	-0.079
Physical pain (anchored)				
2	-0.044	-0.081	-0.068	-0.081
3	-0.111	-0.144	-0.112	-0.136
4	-0.294	-0.283	-0.304	-0.250
5	-0.373	-0.441	-0.433	-0.405
Duration (unanchored)	0.616	1.099	0.451	0.548
Observations	16,218	15,548	16,458	15,600
Log-likelihood	-4544.945	-4393.045	-4919.716	-4640.015
Pseudo R-squared	0.191	0.181	0.139	0.143
Utility of health state with all items at level 2	0.686	0.822	0.699	0.598
Utility of health state with all items at level 3	0.448	0.556	0.599	0.488
Utility of health state with all items at level 4	-0.179	-0.083	-0.089	-0.025
Worst health state utility	-0.634	-0.791	-0.588	-0.539

*Notes*: * The level is not significant at 5%; ** the level is not significant at 10%

^1^ the attribute level is monotonic if its disutility is larger than the better level. For the second level (level 2), the disutility should be negative. All of the non-monotonic or positive disutility are counted

Model explanation: Cohort 1: health-first DCE_TTO_ design with UK sample; Cohort 2: wellbeing-first DCE_TTO_ design with UK sample; Cohort 3: health-first DCE_TTO_ design with Australian sample; Cohort 4: wellbeing-first DCE_TTO_ design with Australian sample

**Table 4:** Wald test with coefficients

	Australian sample			UK sample		
	Level 3	Level 4	Level 5	Level 3	Level 4	Level 5
Seeing	2.71*	0.17	2.31	0.10	2.15	0.34
Hearing	1.45	0	1.51	0.01	1.38	9.42**
Mobility	1.09	1.93	4.20**	2.15	0.38	6.78**
Day to day activities	0.62	0.03	0.41	0.22	0.65	0.73
Sleeping	0.74	1.56	0.04	4.22	4.47**	0.69
Exhausted	2.17	0.58	0.65	4.08**	1.74	1.05
Lonely	0.03	0.03	1.35	0.02	3.65*	0.29
Unsupported	0.38	0.12	0.21	2.80*	2.74*	0
Thinking	0.08	4.11	0.02	0.50	3.07*	1.42
Anxious	0.02	0.32	0.37	0.71	0.20	0.71
Depression	0.10	0.17	3.46*	0.28	2.08	1.74
Control	0.14	0.15	0.22	0.2	0.42	0.01
Physical pain	0.37	2.65	0.77	0.71	0.51	3.38*

*Notes*: Values given here are Wald test score; P value is the probability of obtaining the Wald test statistic given that the null hypothesis is true, which is compared to the critical value 0.05 and 0.1 to determine if the difference is significant

*: 0.1 > p value > 0.05; **: p value < 0.05

## Electronic supplementary material

Below is the link to the electronic supplementary material.

Supplementary Material 1

Supplementary Material 2
