# Supplementary material for: Valuing health and wellbeing using discrete choice experiment: exploring feasibility, design effect and international preference similarity
Source: Eur J Health Econ. 2025 Jul 30;27(2):339–53. doi: 10.1007/s10198-025-01821-3 (PMC13046674; doi:10.1007/s10198-025-01821-3)
Supplement: Supplementary file 1 — Supplementary Material 1 [file 10198_2025_1821_MOESM1_ESM.docx]

**Appendix I The EQ-HWB measure**

Please note EQ-HWB and EQ-HWB-S are copyright product of EuroQol group. The measure is available through online application. See more about the application process on https://euroqol.org/information-and-support/euroqol-instruments/instruments-in-development/eq-hwb/. This study used a 2022 UK version.

|  |
| --- |

**Supplementary Material 1**

This material is to provide more details for the attribute selection, from the EQ-HWB measure. The attribute selection starts with considering the available EQ-HWB developing and psychometric evidence from the following aspects:

1. Dimensionality: dimensionality assessed the completeness of EQ-HWB dimensional structure. First, the study design and valuation include all of the EQ-HWB-S attributes in the final survey. The nine classifier attributes were agreed to be important, and this valuation study would include them. Second, the study design and valuation incorporate at least one item for each high-level domain and a maximum of one item for each sub-domain to maintain the conceptual integrity of EQ-HWB and minimize the concept overlap.

2. Item performance: item performance aspect highlighted the attribute performance with DCE survey. There was a consensus in the theoretical literature that credible attributes and levels for DCE attributes must be relevant for distinguishing health and consistent with the utility theory. Including attributes should be supported by self-reported objective evidence from beneficiaries to prove its satisfying performance on distinguishing health (possible for trade-off) and minimizing the collinearity in linear utility function (possible for generating significant coefficient). First, EQ-HWB attributes with strong correlation score were highlighted and considered to be excluded from this methodology study. Second, attributes with high ceiling or floor effects were highlighted due to low universality. While it was anticipated that some disease-specific conditions, such as vision, hearing and fatigue, might naturally exhibit low prevalence and a skewed distribution, we expected a more balanced distribution in other generic items. Third, attributes with low general validity were highlighted and considered to be excluded. The EQ-HWB research team generated a score ranging from 1 to 4 to represent the overall validity. Attributes with low overall validity score indicated a relatively poor performance in understanding and response.

3. Stakeholder preference: valuation process aimed to incorporate items with high stakeholder preference, including decision-maker, academic, and public preferences. Attributes with lower support rate to be included will be considered for an exclusion. The evidence is reported by two consultations by the EQ-HWB development research, with a total of 71 stakeholders participating, primarily from the UK (54%) and identifying as academics (65%), and one Patient and Public Involvement and Engagement (PPIE).

4. International and cultural feasibility: the feasibility evidence mainly considered Exploratory Factor Analysis (EFA) and Confirmatory Factor Analysis (CFA) results in different countries. The primary EFA and CFA model, rooted in UK survey data, underwent confirmation through CFA analyses across six countries including Australia. Instances of model fitness concerns within specific domains and related items were excluded as leading to mis-specification of the hypothesized causal relations between latent factors and the observed data.

After considering the evidence and evaluating each attribute, the exclusion decisions have been made for several items in the item selection process: Frustrated, Stigma/belonging, Unsafe, Discomfort severity, and Enjoyable activities were excluded due to significant overlap (strong correlation) and/or unsatisfactory overall validity. The Pain frequency attribute was dropped due to domain overlap, while Discomfort severity was excluded due to sub-domain overlap and correlation with pain severity. The decision to exclude Hopeless was driven by evidence from consultation and correlation results. Memory performed less favorably in terms of correlation. The decision to exclude Support was influenced by indications from both overall validity and stakeholder preference, suggesting potential insufficient evidence for confident inclusion. Coping, memory, and support were excluded based on the consensus from Consultation and PPIE. Self-worth was excluded due to unacceptable results from Confirmatory Factor Analysis (CFA).

We decided to include fourteen attributes in the discrete choice survey: Vision, Hearing, Mobility, Daily activities, Control, Anxiety, Depression, Loneliness, Pain severity, Concentrating/thinking clearly, Support, Sleep, Fatigue, and Discomfort frequency after evaluating the EQ-HWB available published evidence.

Then we conducted a research group discussion and qualitative consultation with potential respondents in the UK. The selection of attributes was reviewed with the project’s leading experts and outside qualitative participants. During these discussions, concerns were raised about the 'discomfort' attribute in the future DCE. Experts noted that it might be overly broad, potentially encompassing a wide range of physical symptoms not covered by other physical items, as well as 'mild pain' symptoms, which would be more appropriately categorized under the 'pain' item. Qualitative interviews with healthcare service and care users supported this view, indicating that the 'discomfort' item might lead to ambiguities under the circumstances of quick decision. Hence, it was concluded that 'discomfort' might be unclearly defined and not suitable for this methodology research.

**Vision, Hearing, Mobility, Daily activities, Control, Anxiety, Depression, Loneliness, Pain severity, Concentrating/thinking clearly, Support, Sleep and Fatigue** attributes were included without any wording and level design change.

The following table provides a more detailed evaluation result of each attribute by each evaluation criteria, and the cut-off point for the re-evaluation of evidence:

|  | Attribute | Dimensionality | | Item performance | | | Stakeholder preference | | | International and cultural | Consultation and qualitative evidence |
| --- | --- | --- | --- | --- | --- | --- | --- | --- | --- | --- | --- |
| Rules |  | Domain | EQ-HWB-S | Correlation | Ceiling and floor | Overall validity | Consultation I | Consultation II | PPIE | CFA feasibility | Expert and focus group participants |
| 1 | Vision | ✓ | 🗶 | ✓ | N/A | **O** | ✓ | ✓ | **O** | ✓ | ✓ |
| 2 | Hearing | ✓ | 🗶 | ✓ | N/A | **O** | ✓ | 🗶 | ✓ | ✓ | ✓ |
| 3 | Mobility | ✓ | ✓ | 🗶  Daily activity | 🗶  Floor effect for both | ✓ | ✓ | ✓ | ✓ | 🗶 | ✓ |
| 4 | Daily activity | **O** | ✓ | 🗶  Mobility | 🗶 | **O** | ✓ | ✓ | ✓ | ✓ | ✓ |
| 5 | Self-Care | ✓ | 🗶 | ✓ | 🗶  Floor effect for both | **O** | ✓ | ✓ | ✓ | ✓ | ✓ |
| 6 | Control | **O** | ✓ | 🗶  Loneliness, Support, Concentrating | **O** | **O** | 🗶 | ✓ | 🗶 | ✓ | ✓ |
| 7 | Coping | **O** | 🗶 | **O** | N/A | **O** | 🗶 | ✓ | 🗶 | 🗶 | ✓ |
| 8 | Memory | ✓ | 🗶 | 🗶  concentration | **O** | ✓ | 🗶 | 🗶 | 🗶 | ✓ | ✓ |
| 9 | Concentrating/ thinking clearly | ✓ | ✓ | ✓ | ✓ | ✓ | ✓ | **O** | ✓ | ✓ | ✓ |
| 10 | Anxious | ✓ | ✓ | 🗶  Loneliness, Support, Concentrating, control | ✓ | ✓ | ✓ | ✓ | ✓ | 🗶 | ✓ |
| 11 | Frustrated | ✓ | 🗶 | 🗶  anxious, control, sad | ✓ | ✓ | ✓ | 🗶 | ✓ | ✓ | ✓ |
| 12 | Sad/depressed | ✓ | ✓ | 🗶  cope, control, | ✓ | **O** | 🗶 | ✓ | ✓ | ✓ | ✓ |
| 13 | Hopeless | ✓ | 🗶 | 🗶  happy | ✓ | ✓ | ✓ | 🗶 | **O** | 🗶 | ✓ |
| 14 | Loneliness | ✓ | ✓ | 🗶  support | ✓ | ✓ | ✓ | ✓ | ✓ | ✓ | ✓ |
| 15 | Support | ✓ | 🗶 | 🗶  Lonely, control, left-out | ✓ | ✓ | 🗶 | ✓ | 🗶 | ✓ | **O** |
| 16 | unsafe | ✓ | 🗶 | ✓ | 🗶  Floor effect for both and ceiling effect | **O** | 🗶 | **O** | ✓ | ✓ | ✓ |
| 17 | Sleep | ✓ | 🗶 | **O** | ✓ | N/A | ✓ | ✓ | ✓ | ✓ | ✓ |
| 18 | Fatigue | ✓ | ✓ | ✓ | ✓ | ✓ | ✓ | ✓ | ✓ | ✓ | ✓ |
| 19 | Stigma/belonging | ✓ | 🗶 | 🗶  Self-worth | ✓ | ✓ | ✓ | 🗶 | ✓ | ✓ | ✓ |
| 20 | Self-worth | ✓ | 🗶 | 🗶  Stigma/belonging | **O** | **O** | ✓ | ✓ | 🗶 | 🗶 | ✓ |
| 21 | Enjoyable activities | ✓ | 🗶 | 🗶  Mobility, daily activity | **O** | ✓ | ✓ | ✓ | ✓ | ✓ | ✓ |
| 22 | Pain severity | 🗶 | 🗶 |  |  |  |  |  |  |  | ✓ |
| 23 | Pain frequency | ✓ | ✓ | ✓ | 🗶  Floor effect for both | ✓ | ✓ | ✓ | ✓ | ✓ | ✓ |
| 24 | Discomfort severity | ✓ | 🗶 | **O** | ✓ | ✓ | ✓ | **O** | ✓ | ✓ | 🗶 |
| 25 | Discomfort frequency | 🗶 | 🗶 | 🗶  Severity with pain | 🗶  Floor effect for both | ✓ | ✓ | **O** | ✓ | ✓ | 🗶 |

Note: 1. 🗶 for negative evidence, ✓ for positive evidence, **O** for mixed or no clear evidence due to item setting.

1. Dimensionality evidence derived from published literature, where **O** for the domain Criterion indicated a factor analysis issue and 🗶 means sub-domain overlap.
2. Item performance Criterion Correlation is from the psychometric test E-QALY item level correlations chart, the “How difficult was it for you to get around inside and outside (using any aids you usually use e.g., walking stick, frame or wheelchair)?” is combined evidence of inside and outside, Self-Care item (correlation 0.92 on average) is not flagged, Coping item shows mixed evidence (positive & negative item in correlation chart).
3. The ceiling and floor effect evidence derived from Table A.3: Summary performance of items for distribution and known group difference[162]. Only UK and Australia data evaluated. 🗶 indicates floor or ceiling effect in UK or/and Australia (“XX effect for both” means the effect identified in the two countries). The attribute is reported as **O** if all of the rest four countries showed floor/ceiling problem.
4. The Overall validity and Consultation I evidence derived from Appendix document E-QALY Item Selection Consultation. Consultation II results was from Appendix E-QALY Classification Item Selection Consultation Survey. More information about E-QALY design, process of Consultation I and II, and the data reported can be found in Chapter 2 or the overview paper[162]
5. The Stakeholder preference PPIE Criterion evidence was derived from E-QALY project patient involvement and result report “The role of patient and public involvement and engagement (PPIE) within the development”. Mixed evidence is generated for the domain of Self-Care, Hopeless (the old expression “nothing to look forward to”). Support and Hopeless got negative feedback.
6. If the consultation I and PPIE shared the same opinion on any item, then it should be strongly considered included or excluded.
7. If the consultation II shared similar opinion with the consultation I, then it should be strongly considered included or excluded. However, due to the item selection rule in consultation II, the single rejection from the consultation II data would be considered as suspicious and would be combined with correlation and bolt-on evidence to consider. The **O** indicated this item is on the edge (n=16) of voting out.

If the CFA feasibility evidence is unacceptable, then an item would be excluded, though this criterion is not applied to the EQ-HWB-S items.

**Supplementary Material 2 qualitative survey material**

Qualitative studies have significantly contributed to our understanding of how participants interpret health factors and consider health state preference elicitation tasks. We have divided the participants by four focus groups. Each group with a balanced age, gender and career.

The research aims to shed light on the most appropriate approaches to presenting health and wellbeing information and anchoring the results onto a zero to one scale. The specific areas of focus include:

- Determining whether respondents can interpret the EQ-HWB and DCE information as expected.
- Discuss the pros and cons of various DCE study design strategies and information presentation methods.
- Other design issues, including warm-up questions, introduction wording and question instructions.

Semi-structured questions were crafted in a natural Discrete Choice Experiment (DCE) information presentation order: attributes interpretation, attribute presentation, decision-making strategy, and rethinking the entire design.

Each topic consisted of two or three questions, accompanied by several follow-up sub-questions contingent on participant responses. Digital equipment was utilized for design presentations and discussions. Participants were required to complete the EQ-HWB (selected 13 attributes) questionnaire before participating in the focus groups. After introducing the DCE and research aims, the research host (Haode Wang) led the discussion and summarized the main points of each respondent after their round of speaking. The topics discussed in each focus group are:

Warm-up Question: the interpretation of EQ-HWB questions and DCE as a survey format

Question I: comparison of different DCE designs (Efficient design and partial comparison and order of information)

Question II: how many question and the less common health states

Question III: importance of introduction, understanding what to do, others

All qualitative data sets were analyzed in NVivo 12. The analysis followed a thematic analysis framework. The analysis involved merging the data into a single dataset since all data originated from the same sample group. The data were coded, and a thematic framework was established to capture the interaction between respondents and the presented information. There was no individual-level analysis for the emerged themes and personal characteristics; rather, the group was considered as a whole, and codes were merged to reflect a general attitude during the analysis stage. Thematic analysis involved coding group attitudes on each topic and their underlying reasons. If participants expressed disagreement, that disagreement was coded and classified under other themes.

**Supplementary Material 3 DCE survey design template**

A. Health-first DCE_TTO_

|  | **Life A** |  | **Life B** |
| --- | --- | --- | --- |
| **In seeing** | No difficulty |  | Some difficulty |
| **In hearing** | No difficulty |  | No difficulty |
| **In getting around inside and outside** | Some difficulty |  | Slight difficulty |
| **In doing day-to-day activities** | Slight difficulty |  | Slight difficulty |
| **You have problems with your sleep** | Only occasionally |  | Only occasionally |
| **You feel exhausted** | Only occasionally |  | None of the time |
| **You feel lonely** | Only occasionally |  | Only occasionally |
| **You feel unsupported by people** | Only occasionally |  | Sometimes |
| **You have trouble concentrating/thinking clearly** | Only occasionally |  | Only occasionally |
| **You feel anxious** | Sometimes |  | Often |
| **You feel sad/depressed** | Often |  | Only occasionally |
| **You feel you have no control over your day-to-day life** | None of the time |  | Sometimes |
| **You have physical pain** | Moderate physical pain |  | Moderate physical pain |
| **You will live in the state for** | **4 years and then die** |  | **5 years and then die** |
| **Which would you choose?**  **Life A or Life B** |  |  |  |

B. Wellbeing-first DCE_TTO_

|  | **Life A** |  | **Life B** |
| --- | --- | --- | --- |
| **You feel you have no control over your day-to-day life** | None of the time |  | Sometimes |
| **You feel lonely** | Only occasionally |  | Only occasionally |
| **You feel unsupported by people** | Only occasionally |  | Sometimes |
| **You have trouble concentrating/thinking clearly** | Only occasionally |  | Only occasionally |
| **You feel anxious** | Sometimes |  | Often |
| **You feel sad/depressed** | Often |  | Only occasionally |
| **You have problems with your sleep** | Only occasionally |  | Only occasionally |
| **You feel exhausted** | Only occasionally |  | None of the time |
| **In seeing** | No difficulty |  | Some difficulty |
| **In hearing** | No difficulty |  | No difficulty |
| **In getting around inside and outside** | Some difficulty |  | Slight difficulty |
| **In doing day-to-day activities** | Slight difficulty |  | Slight difficulty |
| **You have physical pain** | Moderate physical pain |  | Moderate physical pain |
| **You will live in the state for** | **4 years and then die** |  | **5 years and then die** |
| **Which would you choose?**  **Life A or Life B** |  |  |  |

**Supplementary Material 4**

**The DCE survey has been designed in the following order:**

Please read the consent form carefully before clicking the consent options

The survey has 3 parts.

1) In Part 1, we will ask you 26 questions about you and your health and wellbeing.

2) In Part 2, we will ask you one practice question plus 13 choice questions.

3) In Part 3, we will ask you some follow-up questions about your decision-making process, attribute importance and what you thought of the survey.

The full survey questionnaire is available through separate document.

**Supplementary Material 5**


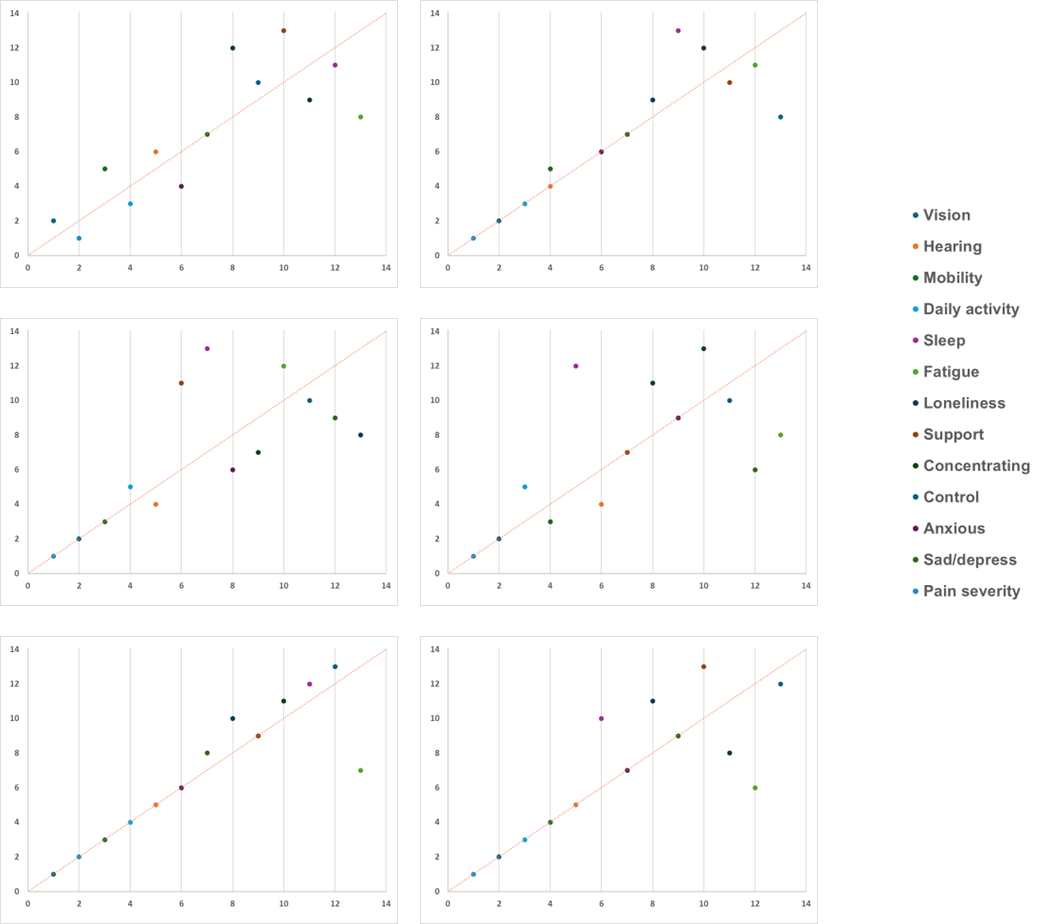
**A. Rank order of stated and modelled preference by models (from top to bottom: Health-first DCE_TTO_ data comparison, Wellbeing-first DCE_TTO_ data comparison) and by country (from left to right: UK and Australian datasets)**

Stated preference rank

Regression Preference Rank

**B. Conditional logit regression with interactive terms**

| Significant interactive terms | UK pooled data | Australian pooled data |
| --- | --- | --- |
| Hearing level 5 (order term) | -0.092 | -0.026 |
| Getting around level 1 (order term) | -0.052 | -0.034 |
| Getting around level 5 (order term) | -0.063 | -0.051 |
| Sleep level 2 (order term) | 0.054 |  |
| Sleep level 4 (order term) | 0.038 | -0.037 |
| Depression level 2 (order term) | 0.072 |  |
| Control level 2 (order term) | -0.061 |  |
| Order main effect | -0.054* | 0.099 |

*Note:** The level is insignificant at 5% but significant at 10%

**
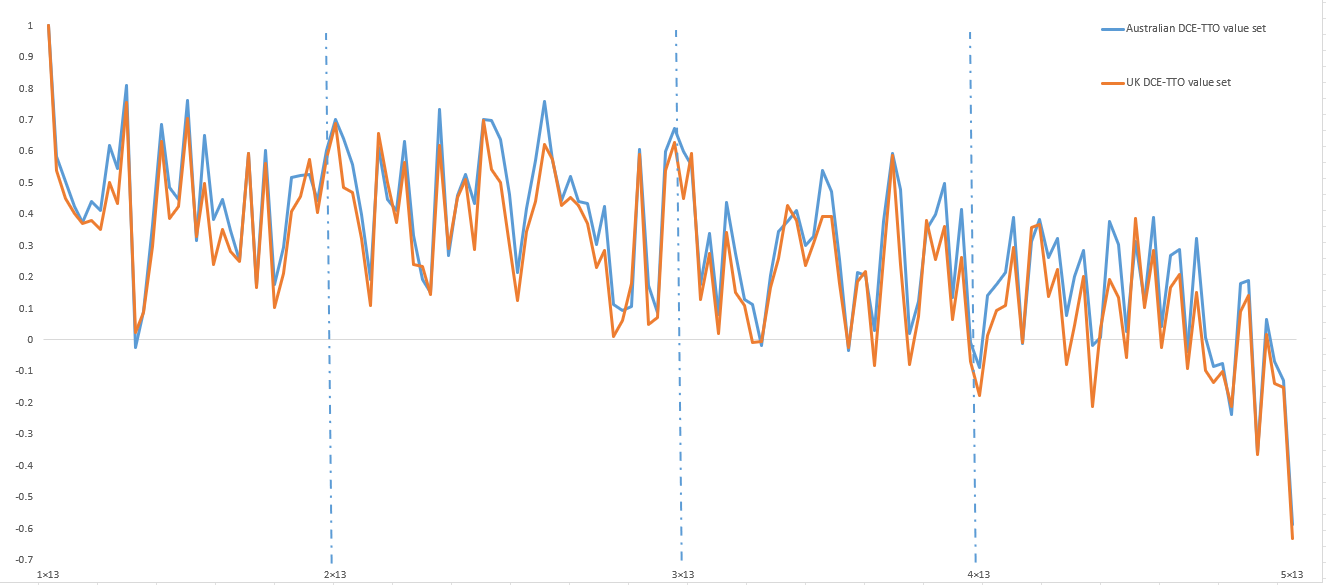
** **Supplementary Material 6: UK and Australia modelled utilities per selected health state (n=244) with DCE_TTO_ health-first design**

Note. Y-axis is the health state utility value. A negative value represents the state has been regarded as worse than death. X-axis is the health states. 1×13 is the health state 1111111111111, 2×13 is the health state 2222222222222, 3×13 is the health state 3333333333333, 4×13 is the health state 4444444444444, 5×13 is the health state 5555555555555. The middle area represents health states in the middle of each benchmark state.
