# Supplementary material for: Valuing health and wellbeing using discrete choice experiment: exploring feasibility, design effect and international preference similarity
Source: Eur J Health Econ. 2025 Jul 30;27(2):339–53. doi: 10.1007/s10198-025-01821-3 (PMC13046674; doi:10.1007/s10198-025-01821-3)
Supplement: Supplementary file 2 — Supplementary Material 2 [file 10198_2025_1821_MOESM2_ESM.docx]

**DCE Survey Template (after giving digital consent)**

**PART 1: Questions about you**

The following questions will ask about you and your health and wellbeing. You should answer all of the questions (select at least one option, including the *Prefer not to say* option) to complete this session.

1. Are you:

Male 🞏

Female 🞏

Other 🞏

Prefer not to say 🞏

2. What is your age (in years):

|  |
| --- |

3. What is your current marital status:

4. Which of the following best describes your main activity? Select one box below.

5. What is the highest level of education you have completed?

6. What is your annual household/individual income before tax (including benefits)?

7. What is your ethnic group?

8. Are you a parent or guardian for a child or children aged under 18 years?

9. Are you a carer for an adult(s) family member or friend (not as a paid job) because of their health or age?

10. Are you cared for by other adults (including paid carers) because of your health or your age?

11. Are your day-to-day activities limited because of a health problem or disability which has lasted, or is expected to last, at least 12 months?

Include problems related to old age.

12. In general, would you say your health is:

13. [Attention check question. **Drop participant** if different answer]

14-26. The following questions will ask about your health and wellbeing in general.

These questions are about the last 7 days.

Please answer all questions. There are no wrong or right answers.

Please select **one response** for each question.

**PART 2: Choosing which life you prefer to live in**

**Survey questions**

*Now we would like you to answer 13 questions. You cannot skip questions or go back to change your answers (you can quit the survey at any time without penalty). Some descriptions are more difficult to imagine than others, please take your time and consider each option carefully. Your choices will be based on your views, there are no right or wrong answers.*

*Please keep going until you finish!*

*[THESE 13 QUESTIONS WILL ALL TAKE THE SAME FORMAT AS THE QUESTION ABOVE, 12 WILL BE FORMAL QUESTIONS AND 1 WILL BE COGNITIVE QUESTION (WITH DOMINATED PAIRS) ]*

**PART 3: After Survey questions:**

**PART 4:** Feedback questions
